# Implications of Heparanase on Heparin Synthesis and Metabolism in Mast Cells

**DOI:** 10.3390/ijms23094821

**Published:** 2022-04-27

**Authors:** Marco Maccarana, Juan Jia, Honglian Li, Xiao Zhang, Israel Vlodavsky, Jin-Ping Li

**Affiliations:** 1 Department of Medical Biochemistry and Microbiology, SciLifeLab, 752 37 Uppsala, Sweden; juanjia1979@yahoo.com (J.J.); honglian.li@imbim.uu.se (H.L.); 2 The Biomedical Center, Department of Medical Cell Biology, University of Uppsala, 752 37 Uppsala, Sweden; xiao.zhang@neuro.uu.se; 3 Cancer and Vascular Biology Research Center, Technion, Haifa 31096, Israel; vlodavsk@mail.huji.ac.il

**Keywords:** antithrombin, heparanase, heparin, mast cells

## Abstract

Heparin is a polysaccharide expressed in animal connective tissue-type mast cells. Owing to the special pentasaccharide sequence, heparin specifically binds to antithrombin (AT) and increases the inhibitory activity of AT towards coagulation enzymes. Heparin isolated from porcine intestinal mucosa has an average molecular weight of 15 kDa, while heparins recovered from rat skin and the peritoneal cavity were 60–100 kDa and can be fragmented by the endo-glucuronidase heparanase in vitro. In this study, we have examined heparin isolated from in vitro matured fetal skin mast cells (FSMC) and peritoneal cavity mast cells (PCMC) collected from wildtype (WT), heparanase knockout (Hpa-KO), and heparanase overexpressing (Hpa-tg) mice. The metabolically ^35^S-labeled heparin products from the mast cells of WT, Hpa-KO, and Hpa-tg mice were compared and analyzed for molecular size and AT-binding activity. The results show that PCMC produced heparins with a size similar to heparin from porcine intestinal mast cells, whilst FSMC produced much longer chains. As expected, heparanase overexpression resulted in the generation of smaller fragments in both cell types, while heparins recovered from heparanase knockout cells were slightly longer than heparin from WT cells. Unexpectedly, we found that heparanase expression affected the production of total glycosaminoglycans (GAGs) and the proportion between heparin and other GAGs but essentially had no effect on heparin catabolism.

## 1. Introduction

Heparin is a linear polysaccharide synthesized by animal connective tissue-type mast cells. The heparin chains are synthesized by the formation of repeating disaccharides, hexuronic acid (HexUA), and D-glucosamine (GlcN) units. During the polymerization process, the sugar units are modified by N-deacetylation/sulfation, C6- and 3-O-sulfation of the GlcN residue, and epimerization/2-O-sulfation of the HexUA residue, resulting in a highly sulfated polymer [1]. The polysaccharide heparin chains are attached to the core protein, serglycin, forming heparin-proteoglycan that is stored in the granules of mast cells. Serglycin can carry either heparin or chondroitin/dermatan sulfate chains (CS/DS) depending on the tissue of origin and location of mast cells that show considerable heterogeneity [2].

Though heparin displays a strong anticoagulation activity in vitro, its physiological implications with regard to hemostasis remain unclarified since heparin-producing mast cells are located in connective tissues. Heparin has been used as a blood anticoagulant in clinical settings since the 1950’s. This activity of heparin depends on the occurrence of a specific pentasaccharide sequence in the polysaccharide that binds to the proteinase inhibitor antithrombin (AT) with high affinity, enhancing its inhibitory activity towards coagulation enzymes [3]. The heparins for the production of commercial drugs are isolated from porcine intestinal mucosa or bovine lung, having an average molecular weight of about 15 kDa. However, heparin molecules isolated from rat skin [4] or rat peritoneal mast cells [5] displayed a molecular size of 60–100 kDa that can be degraded in vitro by incubation with heparanase [6]. The fragmentation of newly-synthesized macromolecular heparin was demonstrated in cultured mast cells [4,7], and the degradation products were similar in size to commercial porcine intestinal MC heparin.

The present study aimed to find out whether heparanase expression affects heparin/GAG production and catabolism in MC derived from different organs. Thus, heparins were isolated from MC maturated from fetal skin (FSMC) and adult peritoneal cavity cells (PCMC) collected from heparanase knockout (Hpa-KO) and heparanase overexpressing (Hpa-tg) mice along with wildtype (WT) animals. It is found that the heparin chains isolated from WT-FSMC are longer than that from WT-PCMC. As expected, heparins from both FSMC and PCMC overexpressing heparanase are shorter than corresponding WT cells. Interestingly, the PCMC gradually switched from heparin to CS/DS production with prolonged in vitro maturation, which is affected by heparanase expression. Whether this phenomenon is associated with the functions of MC in vivo should be examined, especially concerning the functions of MC in allergic reactions.

## 2. Materials and Methods

### 2.1. Animals and Reagents

Heparanase transgenic (*Hpa*-tg) and knockout (*Hpa*-KO) mice on a C57BL/6 background were generated as described [8,9] and maintained by littermates breeding in the animal facility of the Biomedical Center, Uppsala University, Sweden. Wildtype (WT) C57BL/6 mice were used as a control (Ctr). The experimental protocols were approved by the local ethical authority in accordance with the regulations.

Sulfate-free DMEM (AS31600 cat no. 074-91083P) was acquired from Gibco, Uppsala, Sweden. Superose-6 and -12 10/300, PD-10 columns, and DEAE-Sephacel gel were acquired from Cytiva, Uppsala, Sweden. Chondroitinase ABC was acquired from Sigma (Sigma, Solna, Sweden) (cat. no. C3667). Other reagents are described in the respective sections. Bradford reagent (Biorad cat. No. 5000006, Solna, Sweden) was used for protein quantification, using BSA as standard.

### 2.2. Preparation of FSMC and Isolation of PCMC

The fetal skin-derived mast cells (FSMC) were prepared from embryonic mice (E15.5) essentially as described [10,11]. The cells were matured by culturing for up to 4–6 weeks in RPMI-1640 medium, supplemented with 10% FBS, murine stem cell factor (SCF) and IL-3, both at a final concentration of 20 ng/mL (Peprotech, Peprotech, London, UK). The peritoneal cavity mast cells (PCMC) were collected from PBS lavage of 6–8 months-old mice. The cells were cultivated in DMEM with Glutamax, 10% FBS, PEST 1X, MEM 1X, 2-mercaptoethanol 50 µM, murine SCF and IL-3 at 20 ng/mL. For both FSMC and PCMC, the medium was changed every four days, and the unattached MCs were transferred to new plastic plates to continue the culture (the attached cells were discarded). The density of MC at splitting was adjusted to a maximum of 0.5 × 10^6^ cell/mL. The labeling experiments were performed when the cells showed ≥95% toluidine blue positivity (Supplementary Figure S1A). The genetic identity of the *Hpa*-KO and *Hpa*-tg MCs was confirmed by qRT-PCR (Supplementary Figure S1B). The primers used were: Hpa: FP 5′-3′ TTTGCAGCTGGCTTTATGTGG and RP 5′-3′ GAACACCTGCCTCATCACGA; GAPDH FP 5′-3′ TGTGTCCGTCGTGGATCTGA and RP 5′-3′ TTGCTGTTGAAGTCGCAGGAG.

### 2.3. Purification and Analysis of Heparin from the MCs

Upon maturation, the medium was changed to sulfur-free DMEM, 10% FBS, 1X MEM, 2-mercaptoethanol 50 µM, MCF, and IL-3 at the final concentration of 20 ng/mL. After washing twice with this medium, ^35^S-sulfate (Biotech-IgG, Karlstad, Sweden) was added to a final concentration of 100 µCi/mL. The cells were harvested at variable labeling times, washed with PBS, and lysed. GAG chains were released from the core protein by treatment with 50 mM KOH in the presence of 100 mM sodium borohydride for 16 h at 45 °C and purified with DEAE-Sephadex gel equilibrated in a buffer composed of 50 mM acetate, 6 M urea, 0.2 M NaCl, and 0.1% Triton X-100, pH 4.5. After extensive washing with the same buffer, the DEAE-Sephadex bound materials were eluted with a buffer containing 50 mM acetate and 2 M NaCl, pH 4.5. The elutes were desalted on the PD-10 column and lyophilized. Chondroitin sulfate/dermatan sulfate was degraded by chondroitinase ABC (Sigma, Solna, Sweden) (overnight incubation in 50 mM NH_4_OAc, containing 10 mIU ABC). In some experiments, the degraded CS/DS were removed by purification on the DEAE-Sephadex column operated in the same buffer system as above. The chondroitinase ABC-resistant materials were confirmed to be heparin by analysis on the Superose-6 column after HNO_2_ pH 1.5 degradation [12], resulting mainly in disaccharides (Supplementary Figure S2).

### 2.4. Characterization of Heparin

For the assessment of molecular size, the ^35^S-labeled chondroitinase ABC-resistant materials were analyzed on Superose-6 or Superose-12 gel filtration columns, with a running buffer of 0.2 M ammonium bicarbonate and flow rate at 0.25 mL/min. The eluted fractions were collected every 2 min and counted for radioisotope activity. Unlabeled heparin (15 kDa) was included in each analytical run as an internal standard and detected by the dimethylmethylene (DMMB) reaction method [13].

For the analysis of the antithrombin binding property, the purified heparin was applied to a 1-mL antithrombin-Sepharose column prepared as described [14]. The material was applied at a slow flow rate of 0.03 mL/min for about 1 h, and the column was washed for 10 min with Tris 50 mM pH 7.4, 0.25 M NaCl at 1 mL/min, and eluted for 10 min with Tris 50 mM pH 7.4, 2 M NaCl at a flow rate of 1 mL/min.

### 2.5. In Vitro Heparanase Degradation

Heparanase was purified from the heart of the Hpa-tg mice by affinity chromatography on concanavalin A-Sepharose followed by heparin-Sepharose, according to [15]. Purity is shown in Supplementary Figure S4. The purified heparin samples were incubated with the heparanase preparation in 100 µL of 20 mM phosphate/20 mM citrate pH 5.4, 1 mM CaCl_2_, and 50 mM NaCl at 37 °C. After incubation at the indicated time, the resulting materials were analyzed for molecular size by Superose-6 and antithrombin binding activity (as above).

## 3. Results

### 3.1. Heparin Chains Are Longer in FSMC Compared to PCMC

To investigate the impact of heparanase on heparin biosynthesis and metabolism, we have isolated primary mast cells from three mouse strains, WT (wildtype), Hpa-tg (overexpressing heparanase), and Hpa-KO (knockout of heparanase) by two different approaches, i.e., in vitro maturation of cells collected from embryonic fetal skin (FSMC) or from peritoneal cavity lavage (PCMC) of adult mice. The FSMC were normally collected from 1–2 litters of embryos (5–7 animals per litter), while each batch of PCMC was collected from a single adult mouse. Maturation of both MC types, reached after 3–4 weeks, was confirmed by toluidine blue staining (Supplementary Figure S1). To metabolically label GAGs, 1–10 × 10^6^ matured cells were cultured in a sulfate-free medium containing ^35^S (100 µCi/mL) for 24 h. Metabolically ^35^S-labeled heparin was purified as described in ‘Methods’. The purity of heparin isolated from the cell lysates was confirmed by a nitrous acid (pH 1.5) treatment, resulting mainly in ^35^S-labeled disaccharides (Supplementary Figure S2). The potential presence of a trace amount of heparan sulfate (HS) in the samples cannot be excluded but can only be minimal given the similar elution pattern on an anion exchange column of labeled heparins and unlabeled commercial heparin as an internal control (Supplementary Figure S3).

**Figure 1:**
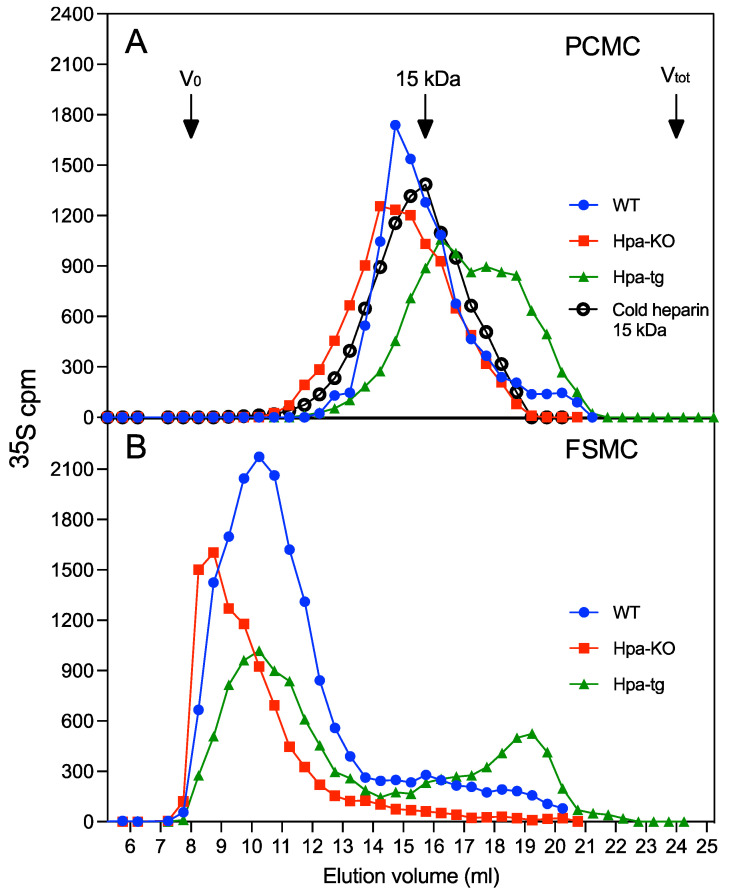
Differential heparin chain length in FSMC and PCMC. Metabolically ^35^S-labeled heparins isolated from PCMC (**A**) and FSMC (**B**) were analyzed on Superose-6 column. Blue circles: WT; red squares: Hpa-KO; green triangles: Hpa-Tg. Unlabeled commercial 15 kDa heparin (empty circles) was analyzed as an internal control. For each genotype the experiment was conducted at three cultivation times performing analytical duplicates. The whole experiment was repeated two times with similar results.

Gel chromatography analysis of the heparins on a Superose-6 column shows that both FSMC and PCMC produced moderately longer heparin chains in Hpa-KO cells and shorter chains in the Hpa-tg cells (Figure 1) in comparison to WT cells. The Hpa-tg heparin was eluted at lower ionic strength on an anion exchange column, indicating that the shorter chains had an overall lower degree of negative charges (Supplementary Figure S3). The heparin samples from both the FSMC and PCMC of Hpa-tg mice contained a fraction of smaller chains that are much shorter than commercial heparin (15 kDa). Notably, the heparins from FSMC are much longer than the heparins from PCMC, regardless of heparanase expression (Figure 1).

### 3.2. Antithrombin Binding of Heparin Is Correlated with Chain Length

**Figure 2:**
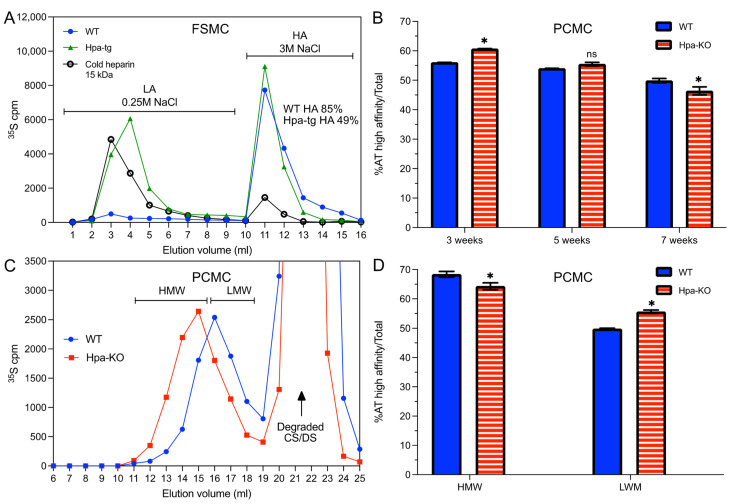
Chain-length and antithrombin binding capacity. (**A**) Metabolically ^35^S-labeled FSMC heparin was applied onto antithrombin (AT)-Sepharose column and eluted with increasing NaCl concentrations. Blue circles: WT; green triangles: Hpa-Tg; black circles: commercial heparin. (**B**) Metabolically ^35^S-labeled heparins isolated from PCMC cultivated for three, five, and seven weeks were fractionated on AT-Sepharose affinity column. Filled blue bars: WT; horizontally striped red bars: Hpa-KO. (**C**) Size fractionation of heparin isolated from PCMC cells cultivated for five weeks. The fractions of high molecular weight (HMW) and low molecular weight (LMW) heparin were pooled as indicated. Blue circles: WT; red squares: Hpa-KO. (**D**) The HMW and LMW heparin fractions prepared in (**C**) were fractionated on AT-Sepharose affinity column. Filled blue bars: WT; horizontally striped red bars: Hpa-KO. Values in (**B**,**D**) represent the mean of analytical triplicates plus/minus one standard deviation. Two-tailed, unpaired *t*-test, * *p* < 0.05 Hpa-KO vs. WT within each cultivation time (**B**) or size pooling (**D**). The whole experiment was repeated two times with similar results.

**Figure 3:**
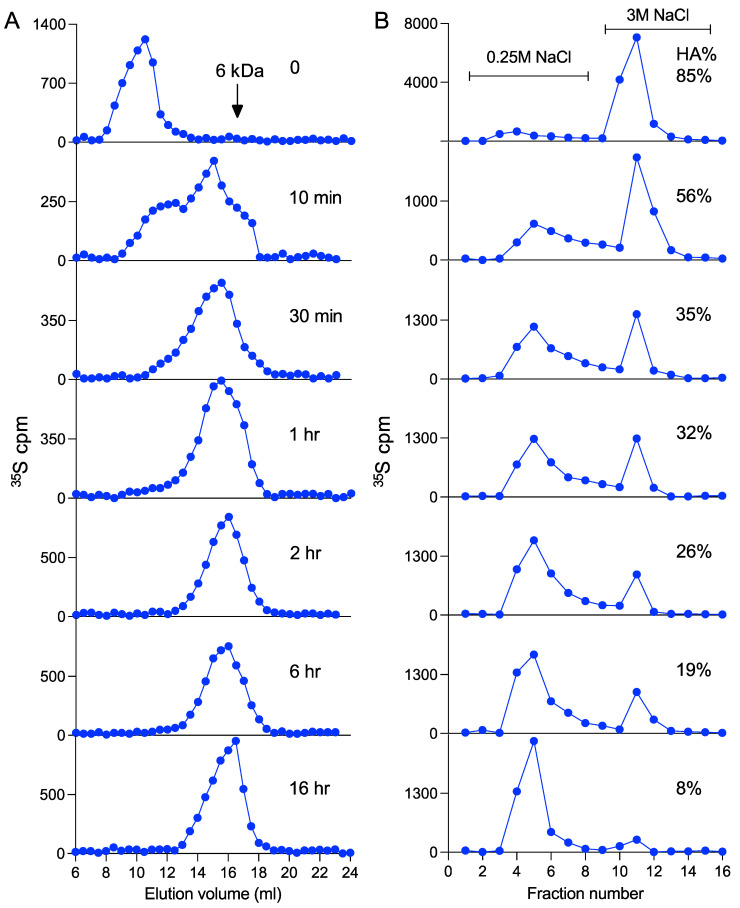
In vitro heparanase degradation of heparin and antithrombin binding. Metabolically ^35^S-labeled WT-FSMC heparin was incubated with the same amount of purified recombinant heparanase (see Supplementary Figure S4 for heparanase preparation) for varied times as indicated in the panels of (**A**). The resultant products were divided into two fractions, one fraction was analyzed for molecular size on Superose-6 (**A**), and the other was applied to AT-Sepharose column eluted with increasing NaCl concentrations (B). The percentage of heparin bound to AT-Sepharose with high affinity (HA) is indicated in each panel of (**B**). Note that the amount of heparin applied onto the columns was different for each chromatography. The values reported in (**B**) represent the mean of analytical duplicates. The whole experiment was repeated two times with similar results.

The commercial porcine intestinal heparin has an average molecular size of 15 kDa, and about 30% of the chains bind to antithrombin (AT) with a high affinity (HA-heparin). To evaluate whether heparanase expression, in addition to the molecular size, has an impact on the AT-binding property of heparin, the purified heparin samples were analyzed on an AT-immobilized Sepharose column. The results revealed that about 85% of the FSMC heparin from WT cells bound to the AT-Sepharose, as compared to only 49% of the Hpa-tg sample (Figure 2A). In comparison, only about 50% of PCMC heparin from WT cells bound to the AT-Sepharose (Figure 2B), and there were minor differences between WT and Hpa-KO heparins from PCMC. It should be noted that the proportion of AT-Sepharose bound heparin was slightly reduced after culturing the PCMC for seven weeks (Figure 2B). The overall recovery of metabolically labeled heparin from Hpa-tg PCMC was low, and the material was not sufficient for the AT-binding analysis (Table 1). The AT-binding capacity is correlated with chain length, as the high molecular weight (HMW) fraction of both WT and Hpa-KO heparin had a higher proportion of AT-Sepharose binding than the low molecular weight (LMW) fractions (Figure 2C,D). The correlation between chain length and AT binding is further demonstrated by the incubation of the WT-FSMC heparin with purified heparanase (Figure 3A). The heparin chains are gradually fragmented by heparanase with increasing incubation time, resulting in an end product of about 6 kDa. Accordingly, the fraction of AT-binding heparin is decreased with a reduction in chain length (Figure 3B).

### 3.3. Heparanase Promotes Shift of PCMC from Heparin to CS/DS Production

Quantification of the total ^35^S-labeled GAGs recovered from cells cultured for four weeks revealed that the total GAGs was about 44,000 cpm/µg protein in the cell lysate of WT-PCMC, while only about 28,000 cpm GAGs/µg protein in the cell lysates of both the Hpa-KO and Hpa-tg-PCMC (Table 1), indicating a reduction in total GAGs synthesis in both transgenic cell types. However, quantification of the total ^35^S-labeled heparin shows a similar level of around 15,000 cpm heparin/µg protein in both WT and Hpa-KO cell lysates. This is due to a higher proportion of heparin versus total GAGs in the Hpa-KO cells, amounting to about 55% in comparison to 32% in WT cells. Heparanase overexpression significantly reduced heparin production to only about 6000 cpm heparin/µg protein, which is about 24% of the total GAGs. Notably, the in vitro maturation shifted the cells from heparin to a more chondroitin-production type with prolonged culturing time, which is apparently correlated with heparanase expression. In WT cells, the percentage of heparin in the cells cultured for four weeks was 32%, while in cells cultured for seven weeks, it reduced to 9%. In comparison, the level of heparin was only moderately reduced in Hpa-KO cells, from 55% to about 45%. Impressively, heparanase overexpression seemingly shifted the majority of the cells to chondroitin expression after culturing for seven weeks, so only about 2% heparin was recovered from Hpa-tg cells.

### 3.4. Heparanase Does Not Affect the Pathway of Heparin Catabolism

**Figure 4:**
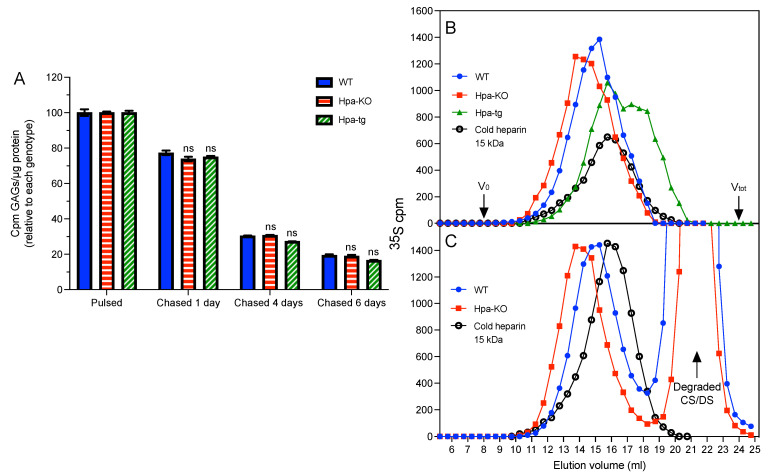
Effect of heparanase on catabolism of GAGs and heparin. (**A**) PCMC cultivated for seven weeks was metabolically labeled with ^35^S for 4 h and then chased for one, four, and six days, respectively. The ^35^S-labeled total GAGs were purified, and the total radioactivity (cpm) was normalized for cellular protein content. Filled blue bars: WT; horizontally striped red bars: Hpa-KO; diagonally striped green bars: Hpa-tg. (**B**) Size analysis (Superose-6 column) of heparins isolated from PCMC immediately after 24 hr labeling and (**C**) after four days chasing. Blue circles: WT; red squares: Hpa-KO; green triangles: Hpa-Tg (note: only a minor amount of heparin was recovered after four-days chasing, which did not allow performance of this analysis). Unlabeled commercial 15 kDa heparin (empty circles) was included as an internal control. Values in (**A**) represent the mean of analytical triplicates plus/minus one standard deviation. Two-tailed, unpaired *t*-test, *ns*, not significant the comparison of Hpa-KO and Hpa-Tg vs. WT within each chasing time. The whole experiment was repeated two times with similar results.

Having seen that heparanase expression altered the synthesis of GAGs and heparin, we wanted to further evaluate the effect of heparanase on the catabolism of GAGs and heparin. The first experiment was performed by labeling the PCMC with ^35^S for 4 h, and chasing for one, four, and six days (after removing ^35^S from the medium). Quantification of the total GAGs, as expected, shows a steady reduction in the total ^35^S-labeled GAGs with chasing (Figure 4A). Unexpectedly, the reduction pattern was similar in all cells, regardless of heparanase expression.

Next, cells were labeled for 24 h, followed by chasing for four days. The isolated heparin was examined by gel chromatography on the Superose-6 column. Again, we found longer heparin chains in Hpa-KO cells and shorter chains in Hpa-tg cells (Figure 4B). Surprisingly, the chromatograms show highly similar elution patterns of heparin before and after chasing, having no degraded fragments (Figure 4C). This suggests a catabolic mechanism of heparin that is fully degraded instead of gradually fragmented, which seems independent of heparanase. Unfortunately, the amount of heparin recovered from the chased Hpa-tg cells was not sufficient for this analysis.

## 4. Discussion

Heparanase was first discovered in mast cells more than 40 years ago [16]. This endo-glucuronidase is the only mammalian enzyme that specifically cleaves the glycosidic bond between glucuronic acid and N-sulfated glucosamine in heparin as well as heparan sulfate (HS) chains. Several reports described substrate recognition properties of heparanase [17,18]. Our earlier study revealed that the enzyme could degrade not only the polysaccharides but also oligosaccharides containing the specific AT-binding pentasaccharide sequence [6]. Based on this property, the chemically synthesized pentasaccharide, fondaparinux, was explored as a substrate for the determination of heparanase enzymatic activity [19].

Heparin isolated from rat skin had an average size of more than 60 kDa [20], while heparins isolated from pig intestinal mucosa or bovine lung have an average molecular weight of 15 kDa. To find out whether specific heparin structures are produced in different types of mast cells, we have characterized the properties of heparin samples isolated from mast cells matured from the skin of embryos (FSMC) and the peritoneal cavity of adult mice (PCMC). Gel chromatographic analysis of metabolically-labeled heparin revealed that FSMC produced significantly longer chains than PCMC. This may be due to a difference between the mast cell subtypes originating from the skin or peritoneal but may also represent a difference between embryonic and adult cells. It has been found that mast cells lacking heparin essentially lost granule content [21], and our study found that several granule proteases are low in Hpa-tg and high in Hpa-KO FSMC [11]. Thus, it is of interest to investigate whether the chain length of heparin is relevant to the functions of mast cells. The results presented here clearly demonstrate a correlation between chain length and the antithrombin binding capacity, which, at least, may be relevant to the anticoagulation activity of heparin. This correlation is rational as binding to antithrombin is dependent on the pentasaccharides that are believed to occur sparsely in long heparin chains. Heparanase degradation generates fragments with and without the pentasaccharide, which are defined as high and low-affinity heparins in binding to antithrombin. It has been believed that each of the high-affinity chains of commercial heparin contained one pentasaccharide; however, a study detected tandem-pentasaccharide in a dodecasaccharide [22].

Heparin is primarily produced in mast cells, but not all mast cells produce heparin. Mast cells show considerable heterogeneity depending on tissue location, and different mast cell subclasses differ substantially in GAG contents [2]. Mucosal-type mast cells (MMC) are predominant in the mucosal layer of the intestine, and in vitro-differentiated mouse bone marrow-derived MC produced only chondroitin sulfate [23]. Connective tissue-type mast cells (CTMC) typically reside in the skin, peritoneal cavity, lung, and intestinal submucosa, mainly producing heparin. The WT-PCMC isolated from adult mice only produced about 30% heparin (meaning 70% CS/DS) of total GAGs (Table 1), and heparin production is further decreased with prolonged culturing. This phenotype shift is seemingly promoted by heparanase, as the knockout of heparanase elevated the proportion of heparin and slowed down the shift. In contrast, overexpression of heparanase led to a rapid decline in heparin production. We cannot distinguish whether this is a cell-type shift or a result of the differential proliferation rate of heparin-producing and CS-producing types of MC. There is no report on whether such a shift occurs in vivo. This finding brings up several functional-related issues. For instance, in addition to the classical roles in an allergic reaction, the important functions of mast cells in other diseases, e.g., cancer, are attracting attention [24]. Do heparin, and CS/DS producing cells have different functions under the conditions of allergy and cancer? Especially since heparanase expression is highly relevant to cancer metastasis [25], does heparanase degradation of heparin in mast cells have an impact on the functions of mast cells in tumor metastasis? Although heparin’s anticoagulation function in vivo remains debatable, our preliminary studies observed an increased amount of heparin in wound areas in a mouse model. This may explain the anticoagulation function of heparin in connective tissue mast cells and functions by inhibiting clot formation in the wound site to promote wound healing through anticoagulation and enhancing the activity of growth factors.

As expected, heparanase overexpression led to the generation of shorter fragments, while the knockout of heparanase resulted in averagely longer chains, regardless of the difference in chain length between FSMC and PCMC (Figure 1). It has been postulated that the long chains of heparin are first fragmented by heparanase in the endosome, then the fragments are translocated to the lysosome to be degraded by exo-glycosidases and sulfatases [7]. To follow the heparin degradation pathway, we set up labeling/chasing experiments, assuming that the fragments of labeled heparin would be accumulated during the chasing period. Unexpectedly, the labeled heparins exhibited a similar pattern of molecular size distribution with and without chasing. This result indicates, for the first time, that the degradation of heparin in MC did not undergo a gradual fragmentation but was rather rapidly and fully degraded by the catabolic enzymes, independent of heparanase. This conclusion is also supported by the finding that heparanase expression did not influence the catabolism rate of total GAGs.

To summarize, heparanase expression affected the chain length of heparin produced by MC and thereby influenced the antithrombin binding capacity and, consequently, the anticoagulant activity. Interestingly, heparanase modulated GAGs and heparin production, a matter that needs to be further investigated. Surprisingly, heparanase expression apparently did not affect the catabolism of heparin in MC.

## Figures and Tables

**Table 1:** Effects of heparanase on production of heparin and total GAGs. PCMC were cultivated for the indicated times. Metabolically ^35^S-labeled GAGs were purified and expressed as total radioactivity (cpm) normalized for cellular protein content. Heparin content was calculated after CS/DS degradation by chondroitinase ABC (see Figure 4C). The data shown are from two independent cell cultivation for each genotype (one cultivation/mouse). Each value presented is the average of analytical duplicates. The whole experiment was repeated twice with similar results.

Genotype	4 Weeks		Heparin/Total GAGs (%)	
	Labeled GAGs (Thousand cpm /µg Protein)	Labeled Heparin (Thousand cpm /µg Protein)	4 Weeks	7 Weeks
WT	44.5 44.1	14.7 14.1	33 32	9 8
Hpa-KO	28.1 28.4	16.3 14.5	58 51	49 41
Hpa-tg	29.0 27.3	6.1 7.1	21 26	2 2

## Data Availability

Not applicable.

## Supplementary Materials

The following supporting information can be downloaded at: https://www.mdpi.com/article/10.3390/ijms23094821/s1.
