# Supplementary material for: Implications of Heparanase on Heparin Synthesis and Metabolism in Mast Cells
Source: Int J Mol Sci. 2022 Apr 27;23(9):4821. doi: 10.3390/ijms23094821 (PMC9102752; doi:10.3390/ijms23094821)
Supplement: Supplementary file 1 [file ijms-23-04821-s001.zip › ijms-1653790-supplementary.pdf]

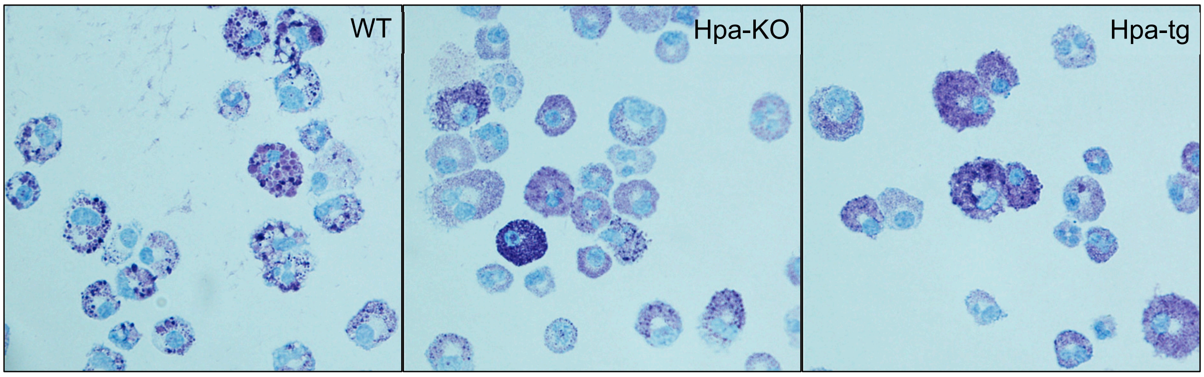

Supplementary Figure S1A. Mast cells were cultivated for 5 weeks and stained with Toluidine blue, indicating the population of matured mast cells.

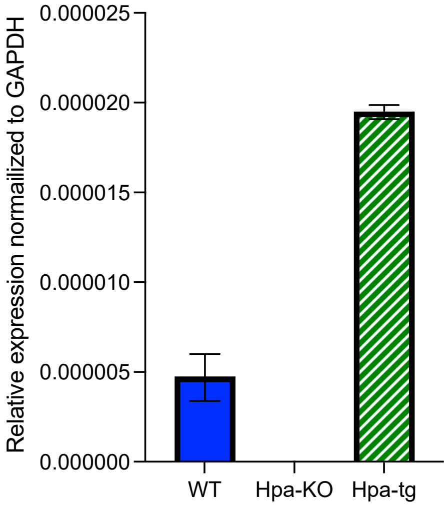

Supplementary Figure S1B. Heparanase expression in PCMC after 4 weeks of cultivation was tested by qRT-PCR.

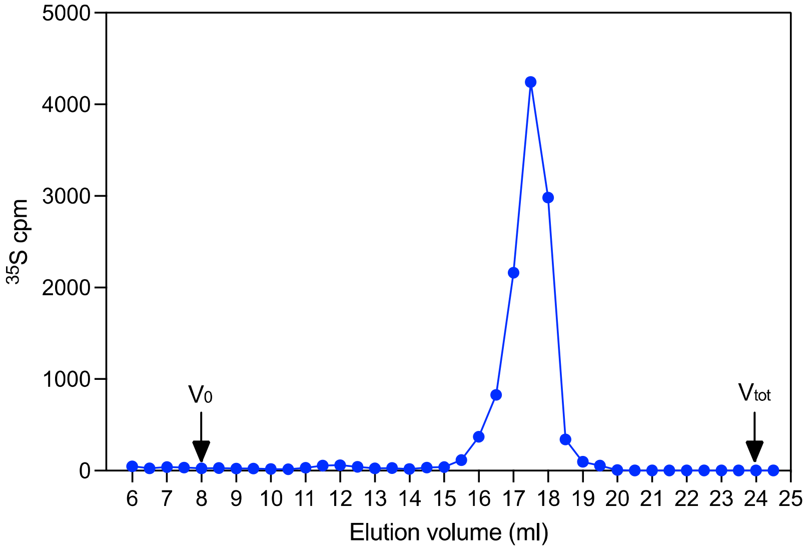

Supplementary Figure S2. Metabolically ^{35}S-labeled FSMC heparin was degraded by deamination cleavage with HNO_2 at pH 1.5 (selective cleavage of the bond between N-sulfated glucosamine and hexuronic acid). The resultant product was analyzed on Superose-12 column. The peak represents generated disaccharides and free sulfates, indicating the purity of the heparin samples.

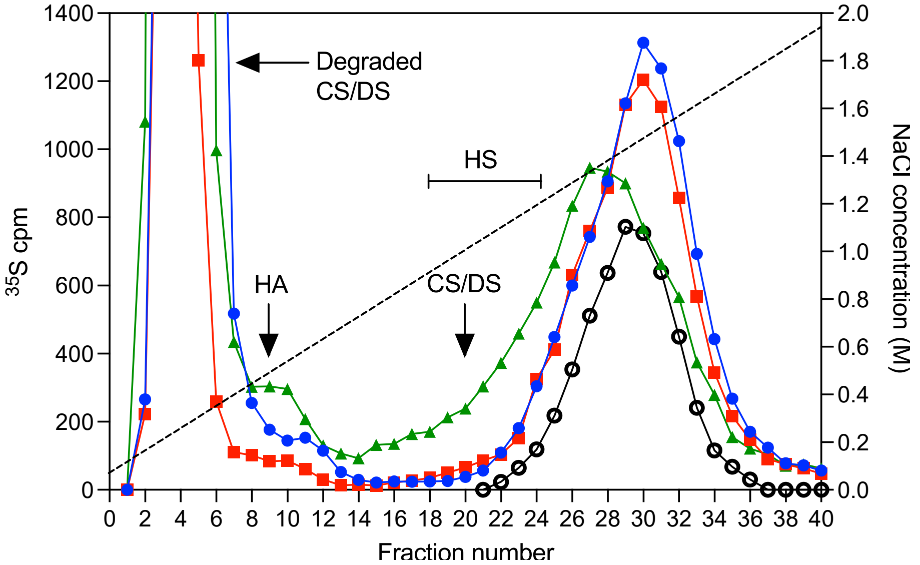

Supplementary Figure S3. Metabolically ^{35}S-labeled PCMC GAGs were treated with chondroitinase ABC and then applied to a DEAE-Sephadex ion-exchange chromatography column eluted with NaCl gradient as indicated. *Blue circles*: WT; *red squares*: Hpa-KO; *green triangles*: Hpa-Tg. Unlabeled commercial 15 kDa heparin (*empty circles*) was co-analyzed as an internal control. Elution positions of HA, CS, and HS are indicated. Heparins from WT and Hpa-KO cells have the same charge density as the commercial heparin, while Hpa-tg heparin had a sub-population with lower charge density. The fractions eluted at lower salt concentrations represent degraded CS/DS disaccharides.

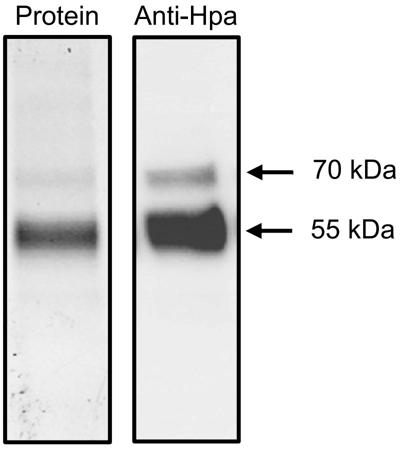

Supplementary Figure S4. Purification of heparanase. Heparanase in lysate from hearts of Hpa-tg mice was purified by concanavalin A-Separose and heparin-Sepharose. (A) SDS-PAGE of the final preparation to show purity (Biorad Stain-free gels). (B) WB anti-heparanase (in-house polyclonal antiserum) to ascertain protein identity.
